# Depressive symptoms and their associations with tuberculosis-related knowledge, attitudes, and practices among patients with multidrug-resistant tuberculosis

**DOI:** 10.3389/fpubh.2026.1851867

**Published:** 2026-06-16

**Authors:** Binh Huy Nguyen, Huy Le Ngoc, Hoa B. Nguyen, Giang M. Le, Luong V. Dinh

**Affiliations:** 1 Hanoi Medical University, National Lung Hospital, Hanoi, Vietnam; 2 Vietnam National Lung Hospital, Hanoi, Vietnam

**Keywords:** attitude, depression, knowledge, multidrug-resistant tuberculosis, PHQ-9, practice

## Abstract

**Objectives:**

To assess the prevalence of depressive symptoms and examine their associations with tuberculosis-related knowledge, attitudes, and practices (KAP) among patients with multidrug-resistant tuberculosis (MDR-TB).

**Methods:**

A cross-sectional study was conducted among 528 MDR-TB patients. Depressive symptoms were measured using the Patient Health Questionnaire-9 (PHQ-9), with a score of ≥10 indicating clinically relevant depression. KAP domains were assessed using a structured scoring system. Associations were analyzed using Spearman correlation and multivariable logistic regression.

**Results:**

The mean PHQ-9 score was 5.32 ± 4.35, and 14.96% of participants (*n* = 79) had clinically relevant depressive symptoms. Among them, 57 had moderate, 17 had moderately severe, and 5 had severe symptoms. Multivariable analysis showed that higher attitude scores were associated with lower odds of depression (aOR = 0.936; 95% CI: 0.886–0.990; *p* = 0.02). Higher practice scores were also strongly associated with reduced depression risk (aOR = 0.837; 95% CI: 0.778–0.901; *p* < 0.001). Knowledge score was not independently associated with depression (*p* = 0.622).

**Conclusion:**

Depressive symptoms are common among MDR-TB patients and are more strongly linked to attitudes and practices than to knowledge alone. These findings suggest the potential value of integrating mental health screening and behavioral support into MDR-TB management programs to improve comprehensive patient care.

## 1 Introduction

Tuberculosis (TB), particularly multidrug-resistant tuberculosis (MDR-TB), remains one of the leading public health challenges globally. According to the World Health Organization, Vietnam continues to be among the countries with the highest burden of TB and MDR-TB worldwide (1). Compared with drug-susceptible TB, MDR-TB requires prolonged treatment regimens (9 to 24 months), the use of second-line drugs with high toxicity, substantial treatment costs, and generally lower success rates. These characteristics make MDR-TB not only a clinical burden but also a severe psychosocial burden for patients (2, 3).

Depression is the most common mental disorder among TB patients, with prevalence rates often markedly higher in MDR-TB due to the prolonged treatment duration, community stigma, economic difficulties, and neurotoxic side effects of the medications (4–6). Clinically, depression reduces motivation and leads to poor treatment adherence. This non-adherence is the main cause of treatment failure, increased mortality risk, and further transmission of drug-resistant strains in the community. Therefore, mental health care is now considered an inseparable component of comprehensive TB care (7, 8).

In addition, knowledge, attitudes, and practices (KAP) regarding TB and MDR-TB play a decisive role in disease management. Correct knowledge helps patients understand transmission mechanisms; positive attitudes reinforce belief in recovery; and correct practices ensure adherence to rigorous treatment regimens. However, real-world studies show a significant “gap” between knowledge and behavior. Depression may act as a mediating factor that exacerbates this gap, making it difficult for patients to translate knowledge into proper practices despite good understanding (9, 10).

In Vietnam, although some studies have examined the psychological status of TB patients, evidence on the specific relationship between depressive symptoms and each KAP component in MDR-TB patients remains limited. Identifying whether depression is more strongly associated with knowledge deficits, negative attitudes, or poor practices is essential. Such data will not only help identify high-risk patients but also provide a basis for designing personalized psychological support and health education interventions, particularly those utilizing information technology for home-based patient management.

The present study is grounded in the Knowledge–Attitudes–Practices (KAP) framework, which posits that knowledge is a necessary but insufficient condition for behavioral change, and that attitudes mediate the translation of knowledge into health-promoting practices (9, 10). Within this framework, depression may function as a psychological barrier that disrupts the knowledge-to-behavior pathway: by reducing self-efficacy, cognitive capacity, and motivation, depressive symptoms may impair patients’ ability to maintain positive treatment-related attitudes and adhere to demanding MDR-TB regimens even when their factual knowledge is adequate (11, 15). Identifying which KAP domain is most closely associated with depressive symptoms therefore has direct implications for the design of targeted psychosocial interventions.

Based on the above reasons, we conducted this study with two specific objectives: (1) to describe the current status of depressive symptoms using the PHQ-9 scale among patients with multidrug-resistant tuberculosis; and (2) to analyze the associations between depressive symptoms and knowledge, attitude, and practice scores regarding TB among the study participants.

## 2 Materials and methods

### 2.1 Study design, setting, and period

This study is a nested cross-sectional analysis conducted within the V-SMART (Vietnam Smartphone-based Medication Adherence for drug-Resistant Tuberculosis) randomized controlled trial. The V-SMART trial enrolled patients with MDR-TB across seven provinces in Vietnam and compared a smartphone application-based intervention with standard directly observed therapy. Full details of the parent trial protocol have been published elsewhere (16). The present analysis focuses on a tuberculosis-related KAP sub-study in which all participants were assessed at a single cross-sectional time point; there was no baseline KAP measurement. Participants in the intervention group (n = 278) were assessed after completing the full MDR-TB treatment course, while participants in the control group (*n* = 250) were assessed during ongoing treatment. This difference in measurement timing between groups is acknowledged as a potential source of systematic bias and is discussed further in the Limitations section. All analyses were conducted at the individual level and study group allocation (intervention vs. control) was adjusted for as a covariate in all multivariable models.

The study was conducted across six provinces of Vietnam, including Hanoi and Ho Chi Minh City, between June 2023 and June 2025. The study was designed to assess depressive symptoms and examine their associations with tuberculosis-related knowledge, attitudes, and practices in this population.

### 2.2 Sample size

Sample size was determined by the parent V-SMART trial protocol, which powered the main trial for the primary treatment adherence outcomes. The current analysis is a secondary cross-sectional analysis of all 528 participants enrolled in the KAP sub-study. A post-hoc power calculation confirmed that this sample provides greater than 90% statistical power to detect a small-to-moderate Spearman correlation coefficient of *ρ* ≥ 0.12 between PHQ-9 scores and KAP domain scores, at a two-sided significance level of *α* = 0.05. For the logistic regression analyses with clinically relevant depressive symptoms (PHQ-9 ≥ 10) as the binary outcome (observed prevalence 14.96%), the sample of 528 participants provides greater than 80% power to detect an adjusted odds ratio of 0.85 or lower per unit increase in practice score at *α* = 0.05.

### 2.3 Participants and recruitment

Eligible participants were identified consecutively from MDR-TB treatment registers at participating outpatient clinics and hospitals across the study provinces. Research staff screened all registered patients meeting eligibility criteria and approached them in the order they appeared on the treatment register (consecutive sampling). Participants were approached by trained independent research staff, who explained the study objectives and procedures and invited them to participate. Data were collected through structured telephone interviews. Because the questionnaire included app-usage questions that were assessed only of the intervention group, interviewers were not blinded to participants’ study arm allocation. This lack of blinding is acknowledged as a potential source of measurement bias.

### 2.4 Inclusion and exclusion criteria

Participants were eligible for inclusion if they were aged 18 years or older, had a confirmed diagnosis of MDR-TB, and were able to provide informed consent and complete the telephone interview. Patients were excluded if they were unable to participate reliably because of severe cognitive impairment, acute medical instability, psychiatric emergency, or any other condition that precluded completion of the questionnaire. Participants with missing data on the primary outcome or key explanatory variables were excluded from the corresponding complete-case analyses.

### 2.5 Measures

#### 2.5.1 Depressive symptoms

Depressive symptoms were assessed using the Vietnamese version of the Patient Health Questionnaire-9 (PHQ-9), a validated 9-item self-report screening instrument for depressive symptom severity. Each item is scored from 0 to 3, yielding a total score ranging from 0 to 27, with higher scores indicating more severe depressive symptoms. For descriptive purposes, PHQ-9 scores were categorized as minimal (0–4), mild (5–9), moderate (10–14), moderately severe (15–19), and severe (20–27). A cutoff score of 10 or higher was used to define clinically relevant depressive symptoms. Participants scoring 15 or higher on the PHQ-9 (moderately severe or severe symptoms, *n* = 22) were flagged during data collection, and their information was shared with the clinical team at the treating facility for follow-up assessment, in accordance with the study’s duty-of-care obligations.

#### 2.5.2 Knowledge, attitudes, and practices regarding MDR-TB treatment

Tuberculosis-related knowledge, attitudes, and practices were assessed using a structured questionnaire specifically developed for this study based on the V-SMART protocol and existing MDR-TB literature. Prior to pilot testing, content validity of the instrument was assessed by a panel of experts from the Vietnam National Tuberculosis Programme (NTP) — who also serve as WHO consultants for tuberculosis control in Vietnam — and tuberculosis specialists from the University of Sydney, Australia. The panel reviewed all items for clinical relevance, comprehensiveness, and cultural appropriateness in the Vietnamese MDR-TB context. The instrument was subsequently piloted in 30 patients with MDR-TB who were not included in the main analysis and was further refined based on their feedback before finalization. A summary of all KAP items, including their content, corresponding dataset variable names, and scoring, is provided in Supplementary Table S1.

The questionnaire comprised three domains. The knowledge domain included 16 items covering the definition of MDR-TB, transmission, risk factors, treatment principles, treatment duration, common adverse effects, and management of side effects. The attitude domain included 11 items scored on a 5-point Likert scale, ranging from strongly disagree to strongly agree, and assessed beliefs about treatment importance, adherence, side-effect reporting, and support-seeking behavior. The practice domain included 12 items assessing self-reported treatment-related behaviors, including medication adherence, missed doses, follow-up attendance, side-effect management, and communication with healthcare providers.

Knowledge score was calculated as the total number of correct responses, with higher scores indicating better knowledge. Attitude score was calculated as the sum of item responses after coding so that higher values reflected more positive treatment-related attitudes. Practice score was calculated as the sum of responses indicating more appropriate or adherent practices, with higher scores reflecting better practices. A total KAP score was also computed by summing the three domain scores, with higher scores indicating more favorable overall knowledge, attitudes, and practices.

Internal consistency of the instrument was satisfactory across domains. Because knowledge items were scored dichotomously (correct/incorrect), the Kuder–Richardson Formula 20 (KR-20) was used as the appropriate reliability coefficient for the knowledge domain (KR-20 = 0.78). Cronbach’s alpha was calculated for the attitude domain (*α* = 0.85) and the practice domain (α = 0.79), as well as for the total KAP score (α = 0.85). All reliability coefficients exceeded the commonly accepted threshold of 0.70, indicating acceptable to good internal consistency.

#### 2.5.3 Other variables

Sociodemographic and clinical characteristics were collected using a structured questionnaire. Variables included age, sex, and study group allocation (intervention vs. control). Additional covariates available for sensitivity analyses included wealth index (a proxy for socioeconomic status), prior tuberculosis treatment history, TB-related stigma (reversed scale), and social support.

### 2.6 Statistical analysis

Data were analyzed using standard statistical procedures. Continuous variables are presented as mean ± standard deviation, and categorical variables are presented as frequencies and percentages. Spearman rank correlation was used to examine associations between PHQ-9 total score and KAP domain scores.

Multivariable logistic regression was performed to identify factors independently associated with clinically relevant depressive symptoms, defined as PHQ-9 ≥ 10. The main independent variables were the knowledge, attitude, and practice domain scores, which were entered simultaneously in the model. Age, sex, and study group allocation were included as covariates on the basis of clinical and methodological relevance. Results are reported as adjusted odds ratios (aOR) with 95% confidence intervals.

To address potential confounding from socioeconomic and clinical factors, a series of sensitivity analyses were conducted by progressively adding available covariates (wealth index, prior tuberculosis treatment history, TB-related stigma, and social support) to the base model. Variance inflation factors (VIF) were calculated to assess multicollinearity. As a pre-specified sensitivity analysis, the multivariable model was repeated restricting the sample to participants currently receiving treatment (*n* = 249), excluding those who had completed treatment. Because study group allocation and treatment status are perfectly collinear in this dataset, stratified analysis by treatment status was not feasible and is not reported; this structural constraint is discussed in the Limitations section.

To account for potential clustering of outcomes within provinces, additional sensitivity analyses were conducted using cluster-robust standard errors with province as the clustering unit (seven clusters: Ho Chi Minh City, An Giang, Can Tho, Hanoi, Thanh Hoa, Tien Giang, and Da Nang). As reliable cluster-robust variance estimation generally requires a larger number of clusters, these results should be interpreted with caution and are presented as supplementary analyses. Analyses were conducted using a complete-case approach. A two-sided *p*-value of less than 0.05 was considered statistically significant. Given the cross-sectional design, all findings were interpreted as associations rather than causal effects.

### 2.7 Ethical considerations

The study was approved by the Human Research Ethics Committee of the University of Sydney (2019/676), the Scientific Council of the Ministry of Science and Technology of Vietnam (08/QĐ-HDQL-NAFOSTED), the Institutional Review Board of the National Lung Hospital of Vietnam (13/19/CT-HDDD), and the Ethics Committee of the National Lung Hospital of Vietnam (VNLH-2023-01). Written informed consent was obtained from all participants prior to data collection. All data were anonymized before analysis and were used solely for research purposes in accordance with the approved study protocols.

## 3 Results

### 3.1 Participant characteristics

A total of 528 patients with MDR-TB were included in the analysis. Socio-demographic and clinical characteristics are presented in Table 1. The mean age was 42.61 ± 13.62 years. Most participants were male (68.75%, *n* = 363), and 30.68% (*n* = 162) were female. Regarding treatment status, 52.65% (*n* = 278) had completed treatment and 47.35% (*n* = 250) were currently receiving treatment. Participants classified as having completed treatment were individuals with a confirmed MDR-TB diagnosis who had successfully finished the full prescribed course of second-line anti-tuberculosis therapy within the V-SMART trial; these individuals are referred to as MDR-TB survivors throughout this paper.

**Table 1:** Socio-demographic and clinical characteristics of the study participants (*N* = 528).

Characteristic	*n*	% or Mean (SD)
Age (years)	528	42.61 (13.62)
Sex	525	
Male	363	68.75
Female	162	30.68
Treatment status		
Completed treatment (MDR-TB survivors)	278	52.65
Currently on treatment	250	47.35

### 3.2 Prevalence and severity of depressive symptoms

The mean PHQ-9 score was 5.32 ± 4.35. According to PHQ-9 severity categories, 264 participants (50.0%) had minimal depressive symptoms, 185 (35.0%) had mild symptoms, 57 (10.8%) had moderate symptoms, 17 (3.2%) had moderately severe symptoms, and 5 (0.9%) had severe symptoms. Overall, 14.96% (*n* = 79) of participants had clinically relevant depressive symptoms (PHQ-9 score ≥ 10).

### 3.3 Descriptive statistics of KAP domain scores

Descriptive statistics for all KAP domain scores are presented in Supplementary Table S2. The mean knowledge score was 9.48 ± 3.23 (possible range 0–16), representing 59.3% of the maximum possible score, with a median of 10.0 (IQR 7.0–12.0). A ceiling effect was negligible for the knowledge domain. The mean attitude score was 33.96 ± 6.25 (possible range 0–44), representing 77.2% of the maximum, with a modest ceiling effect (7.6% of participants achieving the maximum score). The mean practice score was 17.51 ± 2.74 (possible range 0–21), representing 83.4% of the maximum. The mean total KAP score was 60.96 ± 8.62 (possible range 0–81). Compared with participants without clinically relevant depressive symptoms (PHQ-9 < 10, n = 449), those with clinically relevant depression (PHQ-9 ≥ 10, n = 79) had lower mean scores across all KAP domains, with the largest absolute difference observed for practice (17.78 ± 2.45 vs. 16.01 ± 3.71) and total KAP (61.69 ± 8.30 vs. 56.78 ± 9.25).

### 3.4 Associations between depressive symptoms and KAP scores

Spearman correlation coefficients between PHQ-9 scores and the KAP domains are shown in Table 2. The PHQ-9 score was not significantly associated with the knowledge score (*ρ* = 0.012, *p* = 0.845). Depressive symptom severity showed a significant inverse association with the practice score (ρ = −0.275, *p* < 0.001) and the total KAP score (ρ = −0.147, *p* = 0.020). The association with attitude score was inverse but not statistically significant (ρ = −0.057, *p* = 0.369).

**Table 2:** Spearman correlations between PHQ-9 score and tuberculosis-related KAP domains.

Variable	Spearman ρ	*p*-value
Knowledge	0.012	0.845
Attitude	−0.057	0.369
Practice	−0.275	< 0.001
Total KAP score	−0.147	0.020

Correlation matrix analysis showed a moderate positive correlation between knowledge and attitude scores (r = 0.41; *p* < 0.001). However, knowledge was not significantly associated with practice score (*p* = 0.891). Notably, attitude and practice scores showed a weak but significant inverse correlation (r = −0.087; *p* = 0.045). The total KAP score had the strongest positive correlations with attitude (r = 0.840) and knowledge (r = 0.653), while the correlation with practice was the weakest (r = 0.292), although all were statistically significant (*p* < 0.001).

Multivariable logistic regression results are presented in Table 3. Higher attitude scores were independently associated with lower odds of clinically relevant depression (aOR = 0.936; 95% CI: 0.886–0.990; *p* = 0.02). Higher practice scores showed a stronger protective association (aOR = 0.837; 95% CI: 0.778–0.901; *p* < 0.001). The knowledge score was not independently associated with depression (aOR = 0.975; 95% CI: 0.882–1.078; *p* = 0.622). Sensitivity analyses incorporating additional covariates (wealth index, prior tuberculosis treatment, TB-related stigma, and social support) and cluster-robust standard errors did not materially alter the primary estimates; these results are provided in Supplementary Tables S3, S4.

**Table 3:** Multivariable logistic regression analysis of factors associated with clinically relevant depressive symptoms (PHQ-9 ≥ 10).

Variable	aOR	95% CI	*p*-value
Knowledge	0.975	0.882–1.078	0.622
Attitude	0.936	0.886–0.990	**0.02**
Practice	0.837	0.778–0.901	**< 0.001**

aOR, adjusted odds ratio; CI, confidence interval. Model adjusted for age, sex, and study group allocation. Bold *p*-values indicate statistical significance (*p* < 0.05).

## 4 Discussion

This study found a notable burden of depressive symptoms among patients with multidrug-resistant tuberculosis (MDR-TB), with 14.96% of participants meeting the threshold for clinically relevant depressive symptoms on the PHQ-9. Although most participants reported minimal or mild symptoms, a substantial subgroup experienced moderate to severe depressive symptomatology. These findings add to the growing body of evidence that the burden of MDR-TB extends beyond prolonged treatment and physical morbidity to include important psychosocial consequences (14, 12).

A key finding of this study is that depressive symptoms were more closely associated with tuberculosis-related attitudes and practices than with knowledge alone. In the multivariable model, knowledge score was not independently associated with depressive symptoms, whereas higher attitude and practice scores were associated with lower odds of depression. This pattern suggests that factual understanding of the disease, while important, may not be sufficient on its own to buffer psychological distress. Instead, the ways in which patients interpret their illness and engage with treatment-related behaviors may be more closely linked to their mental health status. In settings such as Vietnam, where TB control efforts may have improved general awareness of tuberculosis, persistent psychological burden may be more strongly shaped by emotional and behavioral factors than by deficits in knowledge alone (11, 13).

One possible explanation for this pattern is that attitudes and practices are more proximate to patients’ day-to-day coping experiences during MDR-TB treatment. Positive attitudes may reflect hopefulness, perceived self-efficacy, and trust in treatment, all of which may help reduce feelings of helplessness. Similarly, constructive practices such as medication adherence, attendance at follow-up visits, and active management of side effects may strengthen a sense of personal control over illness. By contrast, knowledge may remain relatively abstract if patients continue to experience stigma, treatment fatigue, social isolation, or uncertainty about recovery. Our correlation findings support this interpretation to some extent: knowledge was moderately correlated with attitude, but was not meaningfully associated with practice. Taken together, these results suggest that knowledge alone does not necessarily translate into adaptive health behaviors, particularly in the context of long, demanding treatment and psychological distress (15).

These findings should be interpreted with appropriate caution because of the cross-sectional design. The direction of the observed associations cannot be determined. Poorer attitudes and practices may contribute to depressive symptoms through reduced engagement, lower confidence, and more difficult treatment experiences (17, 18). Equally, depressive symptoms may lead to more negative attitudes and poorer practices through reduced motivation, impaired concentration, hopelessness, and diminished ability to sustain complex treatment routines (18). It is also plausible that these relationships are mutually reinforcing over time. Longitudinal studies are therefore needed to clarify the temporal and potentially bidirectional relationships between depression and treatment-related attitudes and practices in MDR-TB care (19).

The findings nevertheless have important clinical and policy implications. They suggest that MDR-TB programs may benefit from going beyond knowledge-based education alone and incorporating interventions that address psychosocial and behavioral dimensions of care. Approaches such as motivational counseling, peer support, adherence support, and routine mental health screening may be particularly relevant for patients at risk of poor psychological outcomes. Screening with brief tools such as the PHQ-9 at treatment initiation and during follow-up may help identify patients who require additional support. In high-burden settings, integrating mental health care into patient-centered TB services may improve not only emotional well-being but also treatment engagement and, potentially, downstream clinical outcomes (18, 18, 20).

Our prevalence of clinically relevant depressive symptoms was somewhat lower than that reported in several previous studies of MDR-TB populations in Asia, where higher levels of depression have often been observed (14, 12). However, direct comparison should be made cautiously because differences in study setting, patient selection, treatment phase, and depression measurement may influence prevalence estimates. One possible explanation is that our sample included a higher proportion of patients who were engaged in care or had already developed coping strategies during treatment. Despite these differences in prevalence, our finding that attitudes and practices were more strongly associated with depressive symptoms than knowledge is consistent with broader evidence from low- and middle-income settings suggesting that behavioral and emotional factors may be more closely related to patient well-being and adherence than information alone (11, 17, 21).

This study has several strengths. It included a relatively large sample of patients with MDR-TB, a population that is often difficult to study, and used the PHQ-9, a widely validated screening instrument for depressive symptoms. The KAP instrument underwent content validity review by expert panels from the Vietnam NTP, WHO, and the University of Sydney, and was piloted prior to use. In addition, the separate examination of knowledge, attitude, and practice domains allowed a more nuanced assessment than would have been possible using a single composite measure alone. Sensitivity analyses incorporating all available confounders demonstrated stable primary estimates, supporting the robustness of the findings.

At the same time, several limitations should be acknowledged. First, the cross-sectional design precludes causal inference; the observed associations should not be interpreted as causal. Second, data on specific second-line anti-tuberculosis medications, including agents with potential neuropsychiatric effects such as cycloserine, were not available, and medication-related confounding therefore cannot be excluded (22, 23). Third, several potentially important confounders were unavailable, including educational level, HIV co-infection status, marital status, health insurance coverage, and comorbid conditions. The absence of educational attainment data is a notable limitation, as education may independently influence both depressive symptoms and KAP scores. However, sensitivity analyses incorporating all available socioeconomic and psychosocial proxies demonstrated stable KAP estimates, suggesting that residual confounding, while possible, is unlikely to explain the primary findings. Fourth, a ceiling effect in knowledge scores may have reduced the ability to detect an independent association between knowledge and depressive symptoms. Fifth, the use of self-reported measures introduces the possibility of social desirability and reporting bias. In addition, formal exploratory or confirmatory factor analysis was not conducted for the KAP instrument; future instrument development studies should address this limitation. Sixth, study group allocation and treatment status are perfectly collinear in this dataset, preventing stratified analysis by treatment phase; future studies collecting KAP data at multiple time points within a single group would allow more rigorous examination of how associations change across the treatment trajectory. Future studies should prospectively record total treatment duration in months, given its potential role as a modifier of the relationship between depressive symptoms and KAP domains across different phases of the treatment trajectory. Finally, cluster-robust inference based on seven provinces should be interpreted with caution, as reliable variance estimation generally requires a larger number of clusters (24).

Overall, this study indicates that depressive symptoms among patients with MDR-TB are linked more closely to attitudes and practices than to knowledge alone. The findings support a broader view of MDR-TB management in which mental health, patient beliefs, and treatment-related behaviors are addressed together rather than separately. Strengthening psychosocial support within MDR-TB services may be an important step toward more comprehensive and patient-centered care in high-burden settings.

## 5 Conclusion

In conclusion, depressive symptoms were common among patients with multidrug-resistant tuberculosis in this study, with nearly one in seven participants meeting the threshold for clinically relevant depression. Although tuberculosis-related knowledge was generally high, knowledge alone was not independently associated with depressive symptoms. In contrast, more positive attitudes and better treatment-related practices were consistently associated with lower odds of depression.

These findings suggest that psychological well-being in MDR-TB care may be shaped less by factual understanding alone than by how patients perceive their illness and how they engage with treatment in daily life. Accordingly, MDR-TB programs may benefit from extending beyond knowledge-focused education to include routine mental health screening, counseling, and practical adherence support. Integrating these components into patient-centered TB services may help improve both mental health and treatment engagement in high-burden settings such as Vietnam.

Because of the cross-sectional design, the observed associations should not be interpreted as causal. Nevertheless, the findings indicate an important and potentially actionable association between mental health and behavioral dimensions of MDR-TB care. Future longitudinal studies are needed to clarify the direction of these relationships and to identify interventions that can simultaneously strengthen psychosocial well-being and treatment outcomes.

## Data Availability

The raw data supporting the conclusions of this article will be made available by the authors, without undue reservation.
