# Supplementary material for: Depressive symptoms and their associations with tuberculosis-related knowledge, attitudes, and practices among patients with multidrug-resistant tuberculosis
Source: Front Public Health. 2026 Jun 16;14:1851867. doi: 10.3389/fpubh.2026.1851867 (PMC13351092; doi:10.3389/fpubh.2026.1851867)
Supplement: Supplementary file 1 [file Table_1.DOCX]

**Supplementary Table S1. Summary of KAP questionnaire items, variable names, and scoring**

**SECTION 1: KNOWLEDGE — 17 items, maximum 16 points** *(K4 và K6 cho 0/1/2 điểm tùy partial/full correct; tất cả items còn lại: 0/1)*

| **Item** | **Content (abbreviated)** | **Variable** | **Scoring** |
| --- | --- | --- | --- |
| K1 | Definition of MDR-TB (select one correct definition) | P1.1 | 0/1 |
| K2 | Who can contract MDR-TB? | P1.2 | 0/1 |
| K3 | How is MDR-TB transmitted? | P1.4 | 0/1 |
| K4 | Causes of MDR-TB — non-adherence AND contact with MDR-TB case (both required for full score) | P1.5.1–P1.5.2 | 0/1/2 |
| K5 | Drug combination rationale (select all correct reasons) | P1.7.1–P1.7.2 | 0/1/2 |
| K6 | Main cause of treatment failure | P1.8 | 0/1 |
| K7 | Correct statement about MDR-TB treatment | P1.9 | 0/1 |
| K8 | Treatment duration: 18–24 months (identify correct option) | P1.10 | 0/1 |
| K9 | Common adverse effects — "all of the above" required for full credit | P1.11.1–P1.11.9 | 0/1 |
| K10 | Frequency of adverse event monitoring (monthly = correct) | P1.12 | 0/1 |
| K11 | Ways to manage adverse effects — "all of the above" required | P1.13 | 0/1 |
| K12 | Gastrointestinal complication — identify the INCORRECT statement | P1.15 | 0/1 |
| K13 | Eye abnormality — identify the CORRECT statement (report immediately) | P1.16 | 0/1 |
| K14 | Peripheral neuropathy — identify the INCORRECT statement | P1.17 | 0/1 |
| K15 | Musculoskeletal adverse effect — identify the CORRECT statement | P1.18 | 0/1 |
| K16 | Ototoxicity — identify the CORRECT statement (consult for hearing issues) | P1.19 | 0/1 |

**SECTION 2: ATTITUDE — 11 items, maximum 44 points** *(5-point Likert: Strongly disagree=0 to Strongly agree=4; † reverse-scored: Strongly disagree=4 to Strongly agree=0)*

| **Item** | **Content (abbreviated)** | **Variable** | **Scoring** |
| --- | --- | --- | --- |
| A1 | Completing the full treatment course is important (positive) | TD1 | 0–4 |
| A2 | Adhering strictly to the national protocol is important (positive) | TD2 | 0–4 |
| A3 | I am concerned about treatment efficacy (positive) | TD3 | 0–4 |
| A4 | I can self-manage all side effects without consulting a healthcare worker (NEGATIVE) | TD4 | 0–4 (R) |
| A5 | I would report adverse effects to my healthcare provider immediately (positive) | TD5 | 0–4 |
| A6 | I am willing to take medication at the correct time every day (positive) | TD6 | 0–4 |
| A7 | If I miss a dose, I can take double the next day (NEGATIVE) | TD7 | 0–4 (R) |
| A8 | Taking the correct dose at the correct time is essential (positive) | TD8 | 0–4 |
| A9 | I do not need to report co-medications to my healthcare worker (NEGATIVE) | TD9 | 0–4 (R) |
| A10 | I am confident I can complete the full treatment course (positive) | TD10 | 0–4 |
| A11 | I trust my healthcare team to guide my treatment (positive) | TD11 | 0–4 |

**SECTION 3: PRACTICE — 12 items, maximum 21 points** *(Skip logic applied: if TH1=0, TH2–TH5 set to correct values; if TH8=1, TH9–TH11 set)*

| **Item** | **Content (abbreviated)** | **Variable** | **Scoring** |
| --- | --- | --- | --- |
| P1 | Did you ever miss a dose during treatment? | TH1 | 0/1 |
| P2 | If missed: did you report to your healthcare worker? | TH2 | 0/1 |
| P3 | If missed: what action did you take? (correct = per HCW advice) | TH3 | 0/1 |
| P4 | Did you take all doses on time during treatment? | TH4 | 0/1 |
| P5 | Are you confident about taking medication correctly? | TH5 | 0/1 |
| P6 | Did you ever experience adverse effects? | TH6 | 0/1 |
| P7 | Actions taken when AE occurred (correct = contact HCW or clinic) | TH7.1–TH7.6 | 0/1 |
| P8 | Did you seek medical attention for adverse effects? | TH8 | 0/1 |
| P9 | Reason for not seeking care (skip logic applies) | TH9.1–TH9.6 | 0/1 |
| P10 | What action did you take for adverse effects? (correct = consult HCW) | TH10 | 0/1 |
| P11 | Preferred communication method with healthcare provider | TH11 | 0/1 |
| P12 | What did you do when adverse effects occurred? (multiple scenarios) | TH12.1–TH12.4 | 0/1 |

*Abbreviations: AE = adverse event; HCW = healthcare worker; MDR-TB = multidrug-resistant tuberculosis; R = reverse-scored. † Negative items reverse-scored before summing.*

**Supplementary Table S2. Descriptive statistics of KAP domain scores (N = 528)**

| **Domain** | **Max score** | **Mean (SD)** | **Median (IQR)** | **Min–Max** | **Ceiling n (%)** | **Non-depressed (n=449) Mean (SD)** | **Depressed (n=79) Mean (SD)** | **p-value*** |
| --- | --- | --- | --- | --- | --- | --- | --- | --- |
| Knowledge | 16 | 9.48 (3.23) | 10.0 (7.0–12.0) | 0–16 | 1 (0.2%) | 9.62 (3.28) | 8.71 (2.81) | 0.003 |
| Attitude | 44 | 33.96 (6.25) | 33.0 (29.0–39.0) | 8–44 | 40 (7.6%) | 34.29 (6.07) | 32.06 (6.91) | 0.013 |
| Practice | 21 | 17.51 (2.74) | 18.0 (17.0–19.0) | 3–21 | 28 (5.3%) | 17.78 (2.45) | 16.01 (3.71) | < 0.001 |
| Total KAP | 81 | 60.96 (8.62) | 61.0 (55.0–68.0) | 31–78 | 0 (0.0%) | 61.69 (8.30) | 56.78 (9.25) | < 0.001 |

*Depressed = PHQ-9 ≥ 10 (n=79); Non-depressed = PHQ-9 < 10 (n=449). Ceiling effect defined as achieving the maximum possible domain score. *p-value from Mann–Whitney U test (two-sided). KAP = knowledge, attitudes, and practices; IQR = interquartile range.*

**Supplementary Table S3. Sensitivity analysis: multivariable logistic regression with progressively added confounders (N = 516)**

*Outcome: PHQ-9 ≥ 10 (clinically relevant depressive symptoms). Events: n = 77 (14.9%).*

| **Variable** | **Model 1 Base** | **Model 2 + Wealth index** | **Model 3 + Prior TB treatment** | **Model 4 + Stigma + Social support** | **Model 5 Fully adjusteda** |
| --- | --- | --- | --- | --- | --- |
|  | *aOR (95% CI) / p* | *aOR (95% CI) / p* | *aOR (95% CI) / p* | *aOR (95% CI) / p* | *aOR (95% CI) / p* |
| ***KAP domain scores*** |  |  |  |  |  |
| Knowledge score | 0.947 (0.875–1.024) / 0.169 | 0.941 (0.868–1.020) / 0.141 | 0.946 (0.875–1.024) / 0.168 | 0.948 (0.875–1.026) / 0.185 | 0.942 (0.867–1.023) / 0.155 |
| Attitude score | 0.939 (0.896–0.985) / **0.010** | 0.940 (0.896–0.985) / **0.010** | 0.939 (0.896–0.985) / **0.010** | 0.936 (0.889–0.985) / **0.012** | 0.937 (0.890–0.986) / **0.013** |
| Practice score | 0.851 (0.794–0.913) / **< 0.001** | 0.851 (0.794–0.913) / **< 0.001** | 0.852 (0.794–0.913) / **< 0.001** | 0.851 (0.793–0.913) / **< 0.001** | 0.852 (0.794–0.914) / **< 0.001** |
| ***Sociodemographic*** |  |  |  |  |  |
| Age (years) | 0.993 (0.973–1.013) / 0.476 | 0.993 (0.973–1.013) / 0.474 | 0.993 (0.973–1.013) / 0.480 | 0.993 (0.973–1.013) / 0.477 | 0.992 (0.972–1.013) / 0.476 |
| Female sex | 1.843 (1.065–3.191) / **0.029** | 1.837 (1.071–3.149) / **0.027** | 1.852 (1.080–3.174) / **0.025** | 1.850 (1.068–3.205) / **0.028** | 1.843 (1.065–3.191) / **0.029** |
| Intervention group | 0.638 (0.375–1.087) / 0.099 | 0.638 (0.375–1.085) / 0.097 | 0.643 (0.377–1.099) / 0.106 | 0.629 (0.368–1.074) / 0.091 | 0.680 (0.354–1.304) / 0.246 |
| ***Additional confounders*** |  |  |  |  |  |
| Wealth index | — | 1.038 (0.914–1.179) / 0.569 | — | — | 1.036 (0.912–1.176) / 0.589 |
| Prior TB treatment | — | — | 1.097 (0.586–2.053) / 0.772 | — | 1.099 (0.587–2.055) / 0.769 |
| TB stigma (reversed) | — | — | — | 0.988 (0.911–1.071) / 0.773 | 0.986 (0.909–1.068) / 0.725 |
| Social support | — | — | — | 1.008 (0.943–1.077) / 0.820 | 1.008 (0.943–1.077) / 0.820 |
| ***Model statistics*** |  |  |  |  |  |
| N (complete cases) | 516 | 516 | 516 | 516 | 516 |
| McFadden R² | 0.094 | 0.095 | 0.095 | 0.095 | 0.096 |
| ***VIF (Model 5)b*** |  |  |  |  |  |
| Knowledge | — | — | — | — | 1.41 |
| Attitude | — | — | — | — | 1.54 |
| Practice | — | — | — | — | 1.12 |
| All other variables | — | — | — | — | < 1.60 |

*aOR = adjusted odds ratio; CI = confidence interval; KAP = knowledge, attitudes, and practices; TB = tuberculosis; VIF = variance inflation factor. Bold p-values indicate statistical significance (p < 0.05). For each KAP variable, the first value is aOR (95% CI) and the second is the p-value.* *a Fully adjusted model includes all covariates listed: KAP domain scores, age, sex, study group allocation, wealth index, prior TB treatment history, TB-related stigma (reversed scale), and social support.* *b VIF calculated for Model 5 (fully adjusted). All VIF values < 2.0, indicating no meaningful multicollinearity.*

**Supplementary Table S4. Sensitivity analysis restricted to participants currently receiving MDR-TB treatment (n = 249)**

*Outcome: PHQ-9 ≥ 10 (clinically relevant depressive symptoms). Events: n = 48 (19.3%). McFadden R² = 0.112.* *Note: This stratum includes all control-group participants (currently on treatment). Study group allocation was not included as a covariate because it is constant within this stratum (all participants = control group). Treatment status is therefore not a confounder in this restricted analysis.*

| **Variable** | **aOR** | **95% CI** | **p-value** |
| --- | --- | --- | --- |
| ***KAP domain scores*** |  |  |  |
| Knowledge score | 1.023 | 0.895–1.169 | 0.740 |
| Attitude score | 0.957 | 0.882–1.039 | 0.294 |
| Practice score | 0.819 | 0.746–0.900 | **< 0.001** |
| ***Sociodemographic covariates*** |  |  |  |
| Age (years) | 1.000 | 0.975–1.025 | 0.978 |
| Female sex | 2.971 | 1.490–5.924 | **0.002** |
| ***Model statistics*** |  |  |  |
| N | 249 |  |  |
| Events (PHQ-9 ≥ 10) | 48 (19.3%) |  |  |
| McFadden R² | 0.112 |  |  |

*aOR = adjusted odds ratio; CI = confidence interval; KAP = knowledge, attitudes, and practices; MDR-TB = multidrug-resistant tuberculosis. Bold p-values indicate statistical significance (p < 0.05). Model adjusted for knowledge score, attitude score, practice score, age, and sex. Depression prevalence in this stratum (19.3%) is higher than in the full sample (14.96%), consistent with the greater psychological burden associated with active treatment. The finding that practice score remains the dominant correlate of depression during active treatment, whereas attitude and knowledge are non-significant, is consistent with the primary analysis and supports the robustness of the main conclusions.*
